# Supplementary material for: Environment and density-dependency explain the fine-scale aggregation of tree recruits before and after thinning in a mixed forest of Southern Europe
Source: PeerJ. 2022 Sep 12;10:e13892. doi: 10.7717/peerj.13892 (PMC9477080; doi:10.7717/peerj.13892)
Supplement: Supplementary file 1 [file peerj-10-13892-s001.odt]

Supporting Information.

Javier Rodríguez-Pérez, Bosco Imbert, Javier Peralta. Environment and density-dependency explain the fine-scale aggregation of tree recruits before and after thinning in a mixed forest of Southern Europe. PeerJ.

Appendix S1. Detailed information of material and methods.

|  | Figure S1. Plots and treatments of the thinning intensities (i.e. 0%, 20% and 30% basal-area removed), following a complete randomized block design of nine plots (split-plot). Thinning intensities were applied to the whole plot and  in a buffer area of 5–10 m around them. Data on the spatial distribution of trees and recruits were available for control (i.e. 3, 4 and 9 plots) and 30% thinned plots (i.e. 2, 5 and 7 plots). In the plot inset, we showed the location of the study site (black dot) in the Iberian Peninsula. Image credit: PNOA archive  at [https://datos.gob.es/en/catalogo/e00125901-spaignpnoama](https://datos.gob.es/en/catalogo/e00125901-spaignpnoama). |
| --- | --- |

|  |
| --- |
| Figure S2. Maps of the spatial distribution of trees and recruits in each plot for 2009. In upper panels we show the control plots 3, 4 and 9 (a, b and c panels, respectively), whereas in lower panels the thinned plots 2, 5 and 7 (d, e and f panels, respectively). The size of the circle was proportional to the height of trees and recruits (in log scale). In particular, tree height was calculated from two perpendicular DBH measures (and then averaged) and allometric equations relating the DBH to the height (r = 0.8159 ± 0.0133; mean ± s.e, calibrated for each plot), whereas recruit weight was measured during field sampling. Colours represent species (F. sylvativa (Fs) in red, P. sylvestris (Ps) in green, Q. pubescens (Qh) in blue and Q. ilex (Qi) in purple) whereas fate is in different fills (i.e. alive in empty circles and dead in filled circles). |

|  |
| --- |
| Figure S3. Maps of the species richness of forest canopy (Canopy) observed in 2009. Data derived from field mapping of tree crowns in our study site, which comprised those individual crowns surpassing 2 m in height. To do so, we digitized the canopy areas covering each species to homogeneous areas comprising a single species using QGIS (QGIS Development Team, 2009). For each plot, we accurately mapped the area comprising homogeneous crowns, defined as that those tree crowns assigned to a single species, but comprising one or more tree individuals. For each plot, we computed the species richness of broad-leaf species (i.e. homogeneous areas of broad-leaf species with at least one tree crown); zero values represent pure coniferous areas (i.e. only with Pinus sylvestris one or more tree crowns). Finally, we generated continuous maps at the scale of 1.0 x 1.0 m ranging from mixed coniferous (1.0 values) to pure coniferous (0.0 values). |

| a) Canopy openness |
| --- |
| Figure S4. Maps of the light variables sampled in 2004. Data were obtained from hemispherical photographs (HP) taken in a regular grid of 300 2.0 x 2.0 m cells. For each HP we computed the light variables of canopy openness (CanOpen), Leaf Area Index (LAI; leaf area (m2) / ground area (m2), average diffuse light, both for the whole year (DiffBelow.Yr; Photosynthetic Photon Flux Density) and for the summer period (Diff.Below; PPFD), and number (N.Sunflecks), median duration (Mdn.Sunflecks; seconds) and maximum duration of sunflecks (Max.Sunflecks; seconds) using the Hemiphot.R (ter Steege, 2018) library in R statistical language (R Core Team, 2020). Finally, we generated continuous maps at the scale of 1.0 x 1.0 m using geostatistical methods (kriging). Specifically, we computed an ordinary kriging fitting a spherical variogram model with automap library (Hiemstra et al., 2008) in R statistical language (R Core Team, 2020). |

| b) Leaf Area Index (LAI) c) Average diffuse light for the whole year |
| --- |
| Figure S4. Maps of the light variables sampled in 2004. [Continue] |

| d) Average diffuse light for the summer period e) Number of sunflecks |
| --- |
| Figure S4. Maps of the light variables sampled in 2004. [Continue] |

| f) Median duration of sunflecks g) Maximum duration of sunflecks |
| --- |
| Figure S4. Maps of the light variables sampled in 2004. [Continue] |

| a) Relative abundance of Fagus sylvatica |
| --- |
| Figure S5. Maps of the variables of forest understory sampled in 2008. We only considered those species more abundant and below 2 m in height. For each plot, the relative abundance of plant species were obtained from a regular grid of 300 2.0 x 2.0 m cells. For each sampling point, located in the centroid of each cell, we measured the relative abundance of European beech Fagus sylvatica (Fs), common ivy Hedera helix (Hed), elmleaf blackberry Rubus ulmifolius (Rub), common bracken Pteridium aquilinum (Pter) and neat-feather moss Scleropodium purum (Scl). Finally, we generated continuous maps at the scale of 1.0 x 1.0 m using geostatistical methods (kriging). Specifically, we computed a ordinary kriging fitting a spherical variogram model with automap library (Hiemstra et al. 2008) in R statistical language (R Core Team, 2020). |

| b) Relative abundance of Hedera helix c) Relative abundance of Rubus ulmifolius |
| --- |
| Figure S5. Maps of the variables of understory vegetation sampled in 2008. [Continue] |

| d) Relative abundance of Pteridium aquilinum e) Relative abundance of Scleropodium purum |
| --- |
| Figure S5. Maps of the variables of understory vegetation sampled in 2008. [Continue] |

| a) Density of trees |
| --- |
| Figure S6. Maps of density of trees and recruits of the density-dependent process. Here we only show the  maps of 2009, the first sampling year. Density maps were computed by a kernel smoothed intensity function generated from each point pattern, with a Gaussian kernel with a fixed bandwidth taken as the standard deviation sigma of the isotropic kernel (Diggle, 1985). For computations, we used the spatstat library (Baddeley et al., 2016) in R statistical language. For each plot and year, we computed density-dependent covariates of the density of trees (Dens.adult) and recruits (Dens.rec), the density of the size of recruits (Dens.size.rec), and the density of richness (Dens.rich.rec) and Shannon index of recruits (Dens.shan.rec). In particular, Dens.rich.rec and Dens.shan.rec covariates were calculated from the maps of density of F. sylvatica, Q. pubescens and Q. ilex recruits; in the case of Dens.rich.rec, we used  the median of the intensity of each species as a cut-off value to derive presence/absence maps from (continuous) density maps. |

| b) Density of recruits c) Density of the size of recruits |
| --- |
| Figure S6. Maps of density of trees and recruits of the density-dependent process. [Continue] |

| d) Density of richness e) Density of the Shannon index |
| --- |
| Figure S6. Maps of density of trees and recruits of the density-dependent process. [Continue] |

| a) Environmental variables b) Density-dependent variables |
| --- |
| Figure S7. Cross-correlation between pairs of environmental and density-dependent variables, averaged across studied plots. Colour intensity was proportional to the amount of the Pearson correlation between pairs of variables. Variables were ordered according to the amount of pairwise correlation. For density-dependent variables, we only showed data from 2008. |

Text S1. Summary statistics of spatial-point patterns

The Ripley’s K-function K(r) computes the expected number of points occurring within a distance r from an arbitrary point, which is derived from the intensity function of the point pattern (λ). In geometry terms, K(r) is defined by drawing a circle of radius r centered on an arbitrary point of the pattern. For K(r), the expectation for a random pattern is λπr2 points within r units of the arbitrary point, and thus increases at the rate of r2. Instead of K(r), the L-function L(r) is usually computed due to the expectation for a random pattern increases linearly with r and that improves the visualization and interpretation of the results, compared to K(r). For instance, random patterns yield a value of L(r) = 0, a value of L(r) > 0 at a distance r indicates clustering, and a value of L(r) < 0 indicates a hyper-dispersion.

	The K-function K(r) can be difficult to interpret in terms to the underlying point pattern, as such function is cumulative. In contrast to K(r) function, the pair-correlation function g(r) is a non-cumulative but distance classes are calculated at annuli, not circles, meaning that g(r) only contains information from interpoint distances equal to r (Baddeley et al., 2015). Comparison with the overall point λ intensity g(r) reveals basic properties of the pattern. Specifically, g(r) can be defined by:

g(r) = K’(r) / 2πr

where K’(r) is the derivative of the K(r) with respect to a distance r. Random patterns yields g(r) = 1, which the mean point intensity λ of the point pattern. In addition, clustered patterns yields g(r) > 1 and converges towards 1 with increasing values of r. Regular patterns results in g(r) < 1 for small values, while for larger r, values are greater than 1 (see Illian et al., 2008).

References

Baddeley, A., E. Rubak, and R. Turner. 2015. Spatial point patterns: Methodology and applications with R. CRC Press.

Baddeley, A., R. Turner, and E. Rubak. 2016. Adjusted composite likelihood ratio test for spatial Gibbs point processes. Journal of Statistical Computation and Simulation 86:922–941.

Diggle, P. 1985. A kernel method for smoothing point process data. Journal of the Royal Statistical Society: Series C (Applied Statistics) 34:138–147.

Hiemstra, P. H., E. J. Pebesma, C. J. Twenhöfel, and G. B. Heuvelink. 2009. Real-time automatic interpolation of ambient gamma dose rates from the dutch radioactivity monitoring network. Computers & Geosciences 35:1711–1721.

Illian, J., A. Penttinen, H. Stoyan, and D. Stoyan. 2008. Statistical analysis and modelling of spatial point patterns. John Wiley & Sons.

Law, R., J. Illian, D. F. Burslem, G. Gratzer, C. Gunatilleke, and I. Gunatilleke. 2009. Ecological information from spatial patterns of plants: Insights from point process theory. Journal of Ecology 97:616–628.

QGIS Development Team. 2009. QGIS geographic information system. Open Source Geospatial Foundation.

R Core Team. 2020. R: A language and environment for statistical computing. R Foundation for Statistical Computing, Vienna, Austria.

Steege, H. ter. 2018. Hemiphot. R: Free r scripts to analyse hemispherical photographs for canopy openness, leaf area index and photosynthetic active radiation under forest canopies [online]. Naturalis Biodiversity Center, leiden, netherlands.
