# Supplementary material for: Environment and density-dependency explain the fine-scale aggregation of tree recruits before and after thinning in a mixed forest of Southern Europe
Source: PeerJ. 2022 Sep 12;10:e13892. doi: 10.7717/peerj.13892 (PMC9477080; doi:10.7717/peerj.13892)
Supplement: Supplementary file 2 [file peerj-10-13892-s002.odt]

Supporting Information.

Javier Rodríguez-Pérez, Bosco Imbert, Javier Peralta. Environment and density-dependency explain the fine-scale aggregation of tree recruits before and after thinning in a mixed forest of Southern Europe. PeerJ.

Appendix S2 . Detailed information of the results of point-pattern analyses for plots and treatments

|  |
| --- |
| Figure S1. Analyses of the aggregation of recruits. For each plot, we show a simplified representation of yearly spatial correlograms. The pair correlation functions g(r) from the data are contrasted to simulation envelopes derived from the 199 realizations of the fitted complete spatial randomness (CSR) null models. In lower panels values above simulation envelopes are in green, values below confidence envelopes are in red and values within the envelopes (random) are in white. Upper panels showed control (0%) plots, whereas in lower panels thinned (30%) plots. For a given year, we further computed the goodness of fit (GoF) tests for the intervals of distances of observed data departing from simulation envelopes (distances between gray lines); n.s. p > 0.05; * p < 0.05; ** p < 0.01; and *** p < 0.001. probability values for GoF tests. |

|  |
| --- |
| Figure S2. Analyses of the aggregation of recruits affected by the proximity of trees for control (left panels) and thinned plots (right panels). For each treatment, the results of each of the three plots are averaged. The bivariate g(r) function of the data (black lines) are contrasted to simulation envelopes (grey area) derived from the 199 realizations (i.e. including the 5th lowest and highest values) of the fitted completely random and independently distributed of recruits whereas trees were held fixed.. The expectation of random patterns are as dashed line. In upper plots we show the spatial correlogram of the 2008 year, whereas in lower plots a simplified representation of yearly spatial correlograms. In lower panels values above simulation envelopes are in green, values below confidence envelopes are in red and values within the envelopes (random) are in white. or a given year, we further computed the goodness of fit (GoF) tests for the intervals of distances of observed data departing from simulation envelopes (distances between gray lines); n.s. p > 0.05; * p < 0.05; ** p < 0.01; and *** p < 0.001. probability values for GoF tests. |
|  |

|  |
| --- |
| Figure S3. Analyses of the aggregation of recruits affected by the proximity of trees. For each plot, we show a simplified representation of yearly spatial correlograms. The bivariate g(r) function of the data (black lines) are contrasted to simulation envelopes (grey area) derived from the 199 realizations (i.e. including the 5th lowest and highest values) of the fitted completely random and independently distributed of recruits whereas trees were held fixed. For further conventions, see Fig. S1. |

|  |
| --- |
| Figure S4. Analyses of the survival of recruits affected by the proximity of trees for control (left panels) and thinned plots (right panels). For each treatment, the results of each of the three plots are averaged. The bivariate Lij (r) function of the data (black lines) are contrasted to simulation envelopes (grey area) based on a complete random labelling of recruit survival (i.e. alive or dead) leaving fixed the location of trees. For further conventions, see Fig. S2. |

|  |
| --- |
| Figure S5. Analyses of the survival of recruits affected by the proximity of trees. For each plot, we show a simplified representation of yearly spatial correlograms. The bivariate Lij (r) function of the data are contrasted to simulation envelopes based on a complete random labelling of recruit’s survival (i.e. alive or dead) leaving fixed the location of trees. For further conventions, see Fig. S1. |

Table S1. Summary of the best models fitted for the environmental factors affecting the density of recruits. For each year, the best model was selected according to a stepwise model selection minimising the AIC score. For each model, we represent the estimated of the predicted intensity λ maps, based on linear effects of independent covariates (i.e. environmental factors) on explaining the density of recruits. Values of the covariates represent the coefficients of those included in the best-fitting model. Abbreviations and units of spatial covariates: thinning treatment (Treat; 0% and 30%); the proportion of mixed canopy (Canopy); the averaged diffuse light for the summer period (DiffBelow; Photosynthetic Photon Flux Density); the relative abundance of F. sylvatica (Fs) and Scleropodium purum (Scl) at understory. Non-significant p-values (p > 0.05) and significant variables (p < 0.05) are marked in bold.

a) Summary of best models for 2008

| Variable | Estimate | Std. error | t-value | p-value |
| --- | --- | --- | --- | --- |
| (Intercept) | −6.6764 | 0.4309 | −15.4946 | 0.0000 |
| Treat30% | 1.4522 | 0.3343 | 4.3444 | 0.0000 |
| Canopy | 0.9357 | 0.2360 | 3.9650 | 0.0001 |
| Pter | 1.2032 | 0.5682 | 2.1176 | 0.0342 |
| Hed | 0.8619 | 0.6588 | 1.3083 | 0.1908 |
| Rub | −0.7710 | 0.6417 | −1.2015 | 0.2296 |

b) Summary of best models for 2009

| Variable | Estimate | Std. error | t-value | p-value |
| --- | --- | --- | --- | --- |
| (Intercept) | −6.7241 | 0.4362 | −15.4156 | 0.0000 |
| Treat30% | 1.4653 | 0.3388 | 4.3248 | 0.0000 |
| Canopy | 0.9782 | 0.2396 | 4.0830 | 0.0000 |
| Pter | 1.1850 | 0.5739 | 2.0650 | 0.0389 |
| Hed | 0.9557 | 0.6534 | 1.4626 | 0.1436 |
| Rub | −0.8564 | 0.6512 | −1.3151 | 0.1885 |

c) Summary of best models for 2010

| Variable | Estimate | Std. error | t-value | p-value |
| --- | --- | --- | --- | --- |
| (Intercept) | −6.4504 | 0.5387 | −11.9733 | 0.0000 |
| Treat30% | 1.2537 | 0.3831 | 3.2723 | 0.0011 |
| Canopy | 0.7697 | 0.2798 | 2.7508 | 0.0059 |
| LAI | −1.0609 | 0.7824 | −1.3559 | 0.1751 |
| Pter | 1.7349 | 0.6867 | 2.5267 | 0.0115 |
| Hed | 0.8914 | 0.7728 | 1.1535 | 0.2487 |
| Rub | −1.2985 | 0.7995 | −1.6241 | 0.1044 |
| Scl | 1.1617 | 0.7412 | 1.5673 | 0.1171 |

d) Summary of best models for 2011

| Variable | Estimate | Std. error | t-value | p-value |
| --- | --- | --- | --- | --- |
| (Intercept) | −7.3387 | 0.6403 | −11.4609 | 0.0000 |
| Treat30% | 1.5268 | 0.3713 | 4.1118 | 0.0000 |
| Canopy | 0.9044 | 0.2564 | 3.5280 | 0.0004 |
| DiffBelow | 0.6006 | 0.5586 | 1.0752 | 0.2823 |
| Pter | 0.9439 | 0.6240 | 1.5126 | 0.1304 |
| Hed | 0.7837 | 0.7507 | 1.0440 | 0.2965 |
| Rub | −0.2372 | 0.7241 | −0.3276 | 0.7432 |

Table S2. Summary of the best models fitted for the density-dependent factors affecting the density of recruits. For each year, the best model was selected according to a stepwise model selection minimising the AIC score. For each model, we represent the estimated of the predicted intensity λ maps, based on linear effects of independent covariates (i.e. density-dependent factors) on explaining the density of recruits. Values of the covariates represent the coefficients of those included in the best-fitting model. Abbreviations and units of spatial covariates: thinning treatment (Treat; 0% and 30%); the density of trees (Dens.tree); the size of recruits (Dens.size.rec); the richness (Dens.richn.rec) and the Shannon index (Dens.shan.rec) of the density of recruits. Non-significant u values (p > 0.05) and significant variables (p < 0.05) are marked in bold.

a) Summary of best models for 2008

| Variable | Estimate | Std. error | t-value | p-value |
| --- | --- | --- | --- | --- |
| (Intercept) | −3.2249 | 0.0766 | −42.0783 | 0.0000 |
| Treat30% | −0.1276 | 0.0503 | −2.5338 | 0.0113 |
| Dens.size.rec | 2.4000 | 0.07823 | 0.6781 | 0.0000 |
| Dens.rich.rec | 0.1649 | 0.0379 | 4.3549 | 0.0000 |
| Dens.shan.rec | −0.4430 | 0.1123 | −3.9453 | 0.0001 |

b) Summary of best models for 2009

| Variable | Estimate | Std. error | t-value | p-value |
| --- | --- | --- | --- | --- |
| (Intercept) | −3.3448 | 0.1074 | −31.1568 | 0.0000 |
| Treat30% | 0.1511 | 0.0548 | 2.7556 | 0.0059 |
| Dens.trees | −0.4059 | 0.2092 | −1.9401 | 0.0524 |
| Dens.size.rec | 2.9528 | 0.1189 | 24.8259 | 0.0000 |
| Dens.rich.rec | 0.2043 | 0.0431 | 4.7412 | 0.0000 |
| Dens.shan.rec | −0.6402 | 0.1423 | −4.4986 | 0.0000 |

c) Summary of best models for 2010

| Variable | Estimate | Std. error | t-value | p-value |
| --- | --- | --- | --- | --- |
| (Intercept) | −3.6660 | 0.1079 | −33.9645 | 0.0000 |
| Treat30% | 0.2526 | 0.0609 | 4.1449 | 0.0000 |
| Dens.trees | −0.3912 | 0.2299 | −1.7012 | 0.0889 |
| Dens.size.rec | 3.1730 | 0.1333 | 23.8009 | 0.0000 |
| Dens.rich.rec | 0.2934 | 0.0454 | 6.4636 | 0.0000 |
| Dens.shan.rec | −0.6564 | 0.1404 | −4.6759 | 0.0000 |

d) Summary of best models for 2011

| Variable | Estimate | Std. error | t-value | p-value |
| --- | --- | --- | --- | --- |
| (Intercept) | 0.1092 | −32.4460 | −3.5438 | 0.0000 |
| Treat30% | 0.1755 | 0.0570 | 3.0785 | 0.0021 |
| Dens.trees | −0.4531 | 0.2143 | −2.1144 | 0.0345 |
| Dens.size.rec | 3.2479 | 0.1355 | 23.9659 | 0.0000 |
| Dens.rich.rec | 0.2446 | 0.0457 | 5.3482 | 0.0000 |
| Dens.shan.rec | −0.6268 | 0.1471 | −4.2613 | 0.0000 |

Table S3. Summary of the best models fitted for the environmental factors affecting the density of recruits. For each plot and year, the best model was selected according to a stepwise model selection minimising the AIC score. Acronyms: Ctrl: control, Thnn: thinning, *** p < 0.001, ** p < 0.01, * p < 0.05; values without acronyms, non-significant. For further conventions, see Table S1.

| Plot | Year | Treat | (Intercept) | Canopy | LAI | DiffBelow | Fs | Hed | Rub | Pter | Scl |
| --- | --- | --- | --- | --- | --- | --- | --- | --- | --- | --- | --- |
| 2 | 2008 | Thnn | -2.31 *** | - | - | 0.412 n.s. | - | - | - | 0.701 ** | - |
| 2 | 2009 | Thnn | -2.96 *** | - | - | 0.774 ** | - | - | 0.442 n.s. | 0.511 n.s. | - |
| 2 | 2010 | Thnn | -3.07 *** | -0.237 n.s. | - | 1.025 *** | - | - | - | - | - |
| 2 | 2011 | Thnn | -3.07 *** | - | - | 1.221 *** | -0.729 n.s. | - | - | - | - |
| 3 | 2008 | Ctrl | -1.67 *** | -0.157 n.s. | -0.423 n.s. | - | - | - | - | -0.628 * | - |
| 3 | 2009 | Ctrl | -1.87 *** | -0.182 n.s. | - | - | -0.485 n.s. | - | - | -0.626 * | - |
| 3 | 2010 | Ctrl | -2.33 *** | -0.199 n.s. | - | - | - | - | - | - | - |
| 3 | 2011 | Ctrl | -2.07 *** | -0.281 * | - | - | - | - | - | -0.662 n.s. | - |
| 4 | 2008 | Ctrl | -2.14 *** | -0.809 *** | -0.809 n.s. | 0.606 * | - | - | - | - | -1.825 n.s. |
| 4 | 2009 | Ctrl | -2.93 *** | -0.704 *** | - | 0.479 n.s. | 0.692 n.s. | - | - | - | - |
| 4 | 2010 | Ctrl | -2.76 *** | -0.794 *** | - | - | 0.887 * | - | - | - | - |
| 4 | 2011 | Ctrl | -2.99 *** | -0.823 *** | - | 0.557 n.s. | - | - | - | - | - |
| 5 | 2008 | Thnn | -1.82 *** | 0.336 *** | - | - | - | -0.509 n.s. | - | - | -0.537 * |
| 5 | 2009 | Thnn | -1.92 *** | 0.454 *** | -0.596 ** | - | - | - | - | - | - |
| 5 | 2010 | Thnn | -2.39 *** | 0.423 *** | - | - | - | -0.583 n.s. | - | - | - |
| 5 | 2011 | Thnn | -2.06 *** | 0.462 *** | -0.587 * | - | - | - | - | - | - |
| 7 | 2008 | Thnn | -1.29 *** | 0.230 * | - | -1.060 ** | -0.628 * | - | -1.053 *** | 0.588 * | - |
| 7 | 2009 | Thnn | -1.55 *** | 0.176 n.s. | - | -1.281 ** | - | - | -1.038 *** | 0.601 * | - |
| 7 | 2010 | Thnn | -1.64 *** | 0.212 n.s. | - | -1.201 ** | - | - | -0.838 ** | - | - |
| 7 | 2011 | Thnn | -1.75 *** | 0.200 n.s. | - | -1.233 ** | - | - | -0.861 ** | 0.522 n.s. | - |
| 9 | 2008 | Ctrl | -1.46 *** | -0.439 *** | -0.774 ** | - | - | - | -0.843 *** | 0.825 *** | -1.127 * |
| 9 | 2009 | Ctrl | -1.82 *** | -0.431 *** | -0.824 ** | - | 0.658 * | 0.442 n.s. | -0.723 ** | 0.954 *** | -0.924 n.s. |
| 9 | 2010 | Ctrl | -2.39 *** | -0.452 ** | - | 0.780 ** | 0.546 n.s. | - | -1.139 *** | 1.040 *** | -1.067 n.s. |
| 9 | 2011 | Ctrl | -2.71 *** | -0.445 ** | - | 0.873 *** | 0.952 ** | 0.461 n.s. | -0.684 * | 0.851 ** | - |

Table S4. Summary of the best models fitted for the density-dependent factors affecting the density of recruits. For each plot and year, the best model was selected according to a stepwise model selection minimising the AIC score. Acronyms: Ctrl: control, Thnn: thinning, *** p < 0.001, ** p < 0.01, * p < 0.05; values without acronyms, non-significant. For further conventions, see Table S2.

| Year | Plot | Treat | (Intercept) | Dens.adult | Dens.size.rec | Dens.rich.rec | Dens.shan.rec |
| --- | --- | --- | --- | --- | --- | --- | --- |
| 2008 | 2 | Thnn | -3.12 *** | - | 2.61 *** | 0.173 * | -0.725 ** |
| 2009 | 2 | Thnn | -3.72 *** | - | 4.61 *** | - | -0.457 n.s. |
| 2010 | 2 | Thnn | -4.11 *** | - | 5.17 *** | 0.284 ** | -0.801 ** |
| 2011 | 2 | Thnn | -3.93 *** | - | 5.89 *** | - | -0.558 * |
| 2008 | 3 | Ctrl | -3.33 *** | - | 3.55 *** | - | -0.588 ** |
| 2009 | 3 | Ctrl | -3.56 *** | -1.225 ** | 3.32 *** | 0.147 * | - |
| 2010 | 3 | Ctrl | -3.75 *** | -1.676 *** | 4.59 *** | 0.113 n.s. | - |
| 2011 | 3 | Ctrl | -3.71 *** | -0.887 * | 4.19 *** | - | - |
| 2008 | 4 | Ctrl | -4.26 *** | 0.212 n.s. | 3.44 *** | 0.435 *** | -0.924 ** |
| 2009 | 4 | Ctrl | -4.11 *** | - | 6.55 *** | 0.247 * | -1.251 *** |
| 2010 | 4 | Ctrl | -4.23 *** | - | 8.10 *** | - | -0.847 * |
| 2011 | 4 | Ctrl | -4.13 *** | - | 7.63 *** | 0.269 * | -1.429 ** |
| 2008 | 5 | Thnn | -3.00 *** | - | 2.32 *** | 0.314 *** | -1.006 *** |
| 2009 | 5 | Thnn | -3.32 *** | 0.695 n.s. | 2.96 *** | 0.201 * | -0.786 ** |
| 2010 | 5 | Thnn | -3.57 *** | - | 3.47 *** | 0.393 *** | -0.956 *** |
| 2011 | 5 | Thnn | -3.46 *** | - | 3.43 *** | 0.274 ** | -0.783 ** |
| 2008 | 7 | Thnn | -3.30 *** | - | 2.37 *** | 0.213 ** | -1.219 *** |
| 2009 | 7 | Thnn | -3.13 *** | -1.311 * | 3.16 *** | 0.294 ** | -1.107 *** |
| 2010 | 7 | Thnn | -3.29 *** | -1.019 n.s. | 3.98 *** | 0.165 n.s. | -0.883 ** |
| 2011 | 7 | Thnn | -3.55 *** | -1.229 n.s. | 3.68 *** | 0.327 *** | -0.956 *** |
| 2008 | 9 | Ctrl | -3.03 *** | - | 2.88 *** | - | -0.693 ** |
| 2009 | 9 | Ctrl | -3.10 *** | - | 2.93 *** | - | -0.640 ** |
| 2010 | 9 | Ctrl | -3.53 *** | - | 2.89 *** | 0.229 ** | -0.809 *** |
| 2011 | 9 | Ctrl | -3.03 *** | -0.615 n.s. | 2.91 *** | 0.179 * | -1.015 *** |

|  |
| --- |
| Figure S6. Analyses of the aggregation of recruits affected by environmental factors. For each plot, we show a simplified representation of yearly spatial correlograms. The pair correlation functions g(r) from the data are contrasted to simulation envelopes based on the heterogeneity of environmental covariates within each plot.  For further conventions, see Fig. S2. |

|  |
| --- |
| Figure S7. Analyses of the aggregation of recruits affected by density-dependent factors. For each plot, we show a simplified representation of yearly spatial correlograms. The pair correlation functions g(r) from the data are contrasted to simulation envelopes based on the heterogeneity of environmental covariates within each plot.  For further conventions, see Fig. S2. |

|  |
| --- |
| Figure S8. Values of the predicted intensity function λ of the best model for environmental (left panels) and density-dependent factors (right panels) for each year (panels in each row). Predicted intensity λ values for both factors were extracted from the location of each recruit in each plot, based on the best model of each of the six plots. For each year (in different rows), we grouped recruits based on their survival (red: dead; alive: blue); namely, alive (1) and dead (0) and treatments (x axes of each individual plot). For each factor and survival (dead vs alive), significance between treatments were showed over bars (i.e. Tukey tests including treatment and survival as independent factors). Probabilities: *** p < 0.001, * p < 0.05 and n.s. p > 0.05. |
